# Endothelial‐mesenchymal transition in skeletal muscle: Opportunities and challenges from 3D microphysiological systems

**DOI:** 10.1002/btm2.10644

**Published:** 2024-01-29

**Authors:** Riccardo Francescato, Matteo Moretti, Simone Bersini

**Affiliations:** ^1^ Regenerative Medicine Technologies Laboratory, Laboratories for Translational Research (LRT) Ente Ospedaliero Cantonale (EOC) Bellinzona Switzerland; ^2^ Service of Orthopaedics and Traumatology, Department of Surgery EOC Lugano Switzerland; ^3^ Department of Electronics Information and Bioengineering, Politecnico di Milano Milano Italy; ^4^ Cell and Tissue Engineering Laboratory IRCCS Ospedale Galeazzi ‐ Sant'Ambrogio Milano Italy; ^5^ Euler Institute, Faculty of Biomedical Sciences Università della Svizzera italiana (USI) Lugano Switzerland

**Keywords:** biofabrication, fibrosis, in vitro 3D modeling, organ‐on‐a‐chip, vascularization

## Abstract

Fibrosis is a pathological condition that in the muscular context is linked to primary diseases such as dystrophies, laminopathies, neuromuscular disorders, and volumetric muscle loss following traumas, accidents, and surgeries. Although some basic mechanisms regarding the role of myofibroblasts in the progression of muscle fibrosis have been discovered, our knowledge of the complex cell–cell, and cell–matrix interactions occurring in the fibrotic microenvironment is still rudimentary. Recently, vascular dysfunction has been emerging as a key hallmark of fibrosis through a process called endothelial‐mesenchymal transition (EndoMT). Nevertheless, no effective therapeutic options are currently available for the treatment of muscle fibrosis. This lack is partially due to the absence of advanced in vitro models that can recapitulate the 3D architecture and functionality of a vascularized muscle microenvironment in a human context. These models could be employed for the identification of novel targets and for the screening of potential drugs blocking the progression of the disease. In this review, we explore the potential of 3D human muscle models in studying the role of endothelial cells and EndoMT in muscle fibrotic tissues and identify limitations and opportunities for optimizing the next generation of these microphysiological systems. Starting from the biology of muscle fibrosis and EndoMT, we highlight the synergistic links between different cell populations of the fibrotic microenvironment and how to recapitulate them through microphysiological systems.

**Translational Impact Statement**

No effective therapies are currently available for the treatment of muscle fibrosis, which is a key event in neuromuscular diseases. Traditionally, research on fibrosis has focused on a specific cell type (i.e. myofibroblasts), while neglecting the contribution of the vascular system. More recently, endothelial‐to‐mesenchymal transition has been emerging as a key feature of organ fibrosis. Analyzing the contribution of vascular damage by means of muscle microphysiological systems could lead to the identification of novel druggable targets blocking muscle degeneration during fibrosis.

## 1 INTRODUCTION

Despite the extensive efforts to tackle fibrosis, an effective treatment is still missing.^1^ Indeed, just two drugs are currently available for the treatment of fibrosis being however limited to Idiopathic Pulmonary Fibrosis.^2^, ^3^ For the treatment of muscular fibrosis, Glucocorticoids are the only option that has shown so far efficacy in preserving muscle force and locomotion in dystrophic patients.^4^ However, Glucocorticoids are non‐specific steroids that might have many side effects resulting in a contribution to muscle wasting.^5^ This lack of treatments is particularly dramatic when considering the incidence of fibrosis. Fibrosis is a pathological condition which, in the muscular context, is linked to primary diseases like muscular dystrophies, laminopathies, and neuromuscular disorders,^6^ but also volumetric muscle loss following traumas, accidents, and surgeries.^7^ It is estimated that 1 in 1000 births are cases of dystrophies^8^ and 4.5 million muscular reconstructive surgical operations are performed annually as a result of car accidents, cancer ablation, or cosmetic procedures,^9^ often resulting in the onset of fibrosis.

The lack of effective treatments for fibrosis is due to its complex nature and to the absence of proper models to mimic its onset and progression. The many cellular, biochemical, and biophysical factors involved make it challenging to understand the sequence of events leading to the disease and to find an effective therapeutic solution.^10^ Due to the lack of species‐specificity, animal models may not be the best option to identify new drugs. Conventional 2D in vitro setups, while incorporating human primary cells, are limited in their ability to accurately represent native tissue architecture and functionality.^11^ Thus, there is a need for more physiologically relevant models to efficiently identify novel therapies (such as cell‐based, DNA‐based, or drug‐based therapies).

**FIGURE 1:**
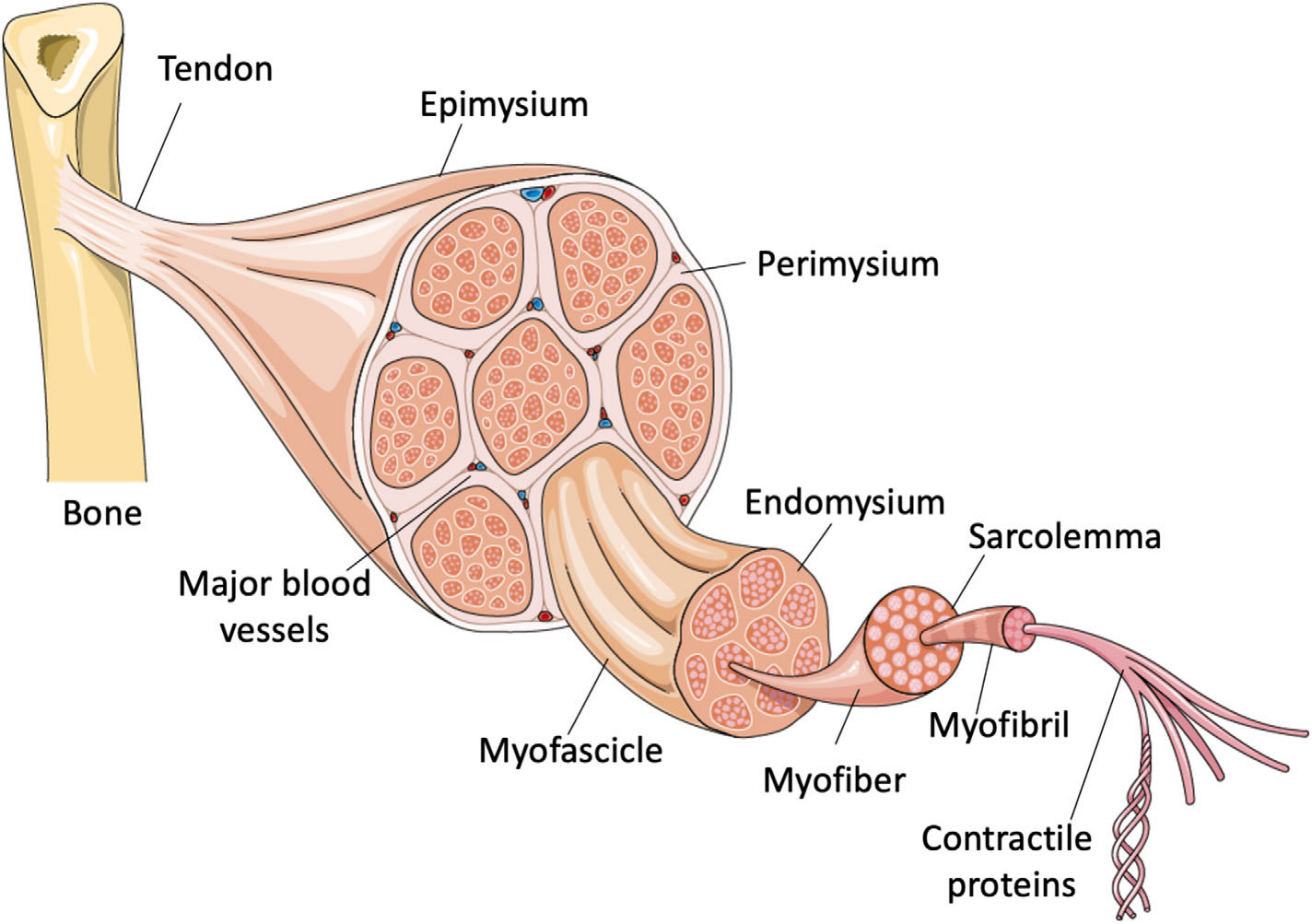
Schematization of the skeletal muscle anatomy. Each myofiber contains numerous myofibrils that are made of contractile proteins. Myofibers are densely packed together forming a myofascicle. Several myofascicles constitute the muscle body. In the perimysium, which surrounds the myofascicles, and in the endomysium, which is located around the myofibers, stromal cells (e.g., vascular cells, motor neurons, fibroblasts, resident macrophages) contribute to the generation of the muscle microenvironment. The Figure was partly generated using Servier Medical Art, provided by Servier, licensed under a Creative Commons Attribution 3.0 unported license.

Advancements in bioengineering and cell biology have led to the development of 3D in vitro models of various tissues and organs, including muscles, which have improved our capacity to reproduce key aspects of native tissues in highly controlled experimental platforms.^12^, ^13^ Although major progress has been made in recent years, 3D muscle models have yet to reach their full potential. By taking advantage of the high experimental control offered by these technologies, it is now possible to start deciphering complex pathological mechanisms underlying muscle fibrosis and other muscle‐related diseases. An emerging hypothesis depicts the endothelium as a key player in the fibrotic process, as it can undergo a transdifferentiation known as endothelial‐mesenchymal transition (EndoMT), leading to the acquisition of a fibrogenic phenotype.^14^ This involvement has been proven in various fibrotic tissues such as the heart, skin, kidney, and lungs. However, EndoMT in muscular fibrosis is poorly understood and under‐investigated. Additionally, most of the studies are based on murine models, which might represent a limitation when species‐specificity plays a role. In this review, we explore the potential of 3D human muscle models in studying the role of endothelial cells (ECs) in muscle fibrosis and identify limitations and opportunities for their optimization. Further research on EndoMT has the potential to lead to the development of new treatments by taking advantage of microphysiological systems mimicking the architecture and functionality of the human muscle microenvironment (Figure 1).

## 2 MUSCULAR FIBROSIS

Fibrosis is a pathological condition that affects various organs, including skeletal muscle, heart, liver, lungs, and kidneys.^1^ It can also occur in other tissues leading to organ failure. The key hallmark of fibrosis is the excessive accumulation of extracellular matrix (ECM), which disrupts the diffusion of biochemical cues^15^, ^16^ and causes changes in local stiffness and biomechanical parameters.^17^, ^18^, ^19^ These changes lead to cellular dysfunction and were traditionally associated with a single cell population, the myofibroblasts. The onset of fibrosis is believed to start with a tissue injury that activates an inflammatory response.^20^ After the injury, ECM is produced as a temporary support for tissue regeneration and the number of ECM‐producing myofibroblasts increases in the area. Usually, the process ends with the decline of myofibroblasts, either through apoptosis or deactivation, and restoration of normal tissue.^21^ However, if myofibroblasts persist, fibrosis develops and leads to the loss of tissue function. In the case of skeletal muscle tissue, this process results in a decline in the ability to generate contractile force.^22^ Myofibroblasts were initially identified as a regenerative phenotype acquired by quiescent fibroblasts.^23^ Both these cell types have poorly characterized phenotypes since there is no known single marker that specifically discriminates fibroblasts or myofibroblasts from any other mesenchymal cell type. Instead, all these cell populations have in common the expression of mesenchymal markers, such as cadherin‐2 (N‐cadherin), cadherin‐11 (OB‐cadherin), fibroblast‐specific protein‐1 (FSP‐1/S1004A), SM22 and Vimentin.^24^ Over time, several other cell populations have been identified as myofibroblast sources thanks to cell lineage tracing studies.^25^ It has been demonstrated that myofibroblast progenitors express Gli1 and ADAM12, like pericytes,^26^ but also PDGFRα,^27^ being a marker of mesenchymal cell types like fibro‐adipogenic progenitors (FAPs).^28^, ^29^ Tie1^+^ bone marrow‐derived progenitor cells were also proven to be a source of fibrotic cells in cardiac fibrosis.^30^ Macrophages have been recently added to the list of sources of myofibroblasts^31^ together with ECs,^32^, ^33^ as discussed in the next paragraphs of the present review.

The chain of events that leads so many different cell populations to develop myofibroblast‐like properties is currently unknown. Understanding the specific local microenvironment characteristics that determine which cells will develop fibrotic properties is crucial in controlling the process and restoring tissue function before it reaches the end stage of fibrosis. For this reason, it is critical to gain insights into how certain cellular sources can act as drivers and recruit other cell populations in a fibrogenic cascade, in order to abort this process and prevent systemic disease. The endothelium is an essential cellular source to investigate in this regard. Indeed, the endothelium has not only been already associated with fibrosis in numerous tissues (e.g. liver, kidney), but it is also homogeneously distributed across the human body. Furthermore, it develops in close contact with muscle fibers, maintaining a two‐way communication with them. For these reasons, vascularized muscle‐specific fibrotic tissue models might be of great help to identify alternative markers of muscle fibrosis and providing more effective therapeutic options.

## 3 THE IMPORTANCE OF ENDOTHELIAL CELLS IN THE SKELETAL MUSCLE

Skeletal muscle has a well‐defined architecture based on bundles of parallel individual myofibers, embedded in an ECM and associated with blood capillaries and nerves. The ECM is divided into three zones: the endomysium, perimysium, and epimysium. The individual myofibers and stromal cells are dispersed in the endomysium, the inner ECM zone, forming a unit named fascicle. Each fascicle is then located in the perimysium, the middle ECM layer that supports numerous fascicles. Finally, the whole system is surrounded by the epimysium, the outer and thicker connective tissue layer.^34^ The skeletal muscle vasculature has a hierarchical organization. Arteries penetrate the epimysium. In the perimysium, they split into a network of interconnected arterioles, which enter the endomysium. There, they branch into a meshwork of capillaries that develop in parallel to the myofibers following the direction of the muscle.^35^ The microcirculation of skeletal muscle is composed of two cell types: ECs and pericytes. ECs form the walls of the capillaries, which are around 200 μm in diameter. Muscle contractions exert strain on the vessel wall. ECs sense and transmit different types of external forces primarily through their cell–cell, and cell–ECM adhesions, which also generate intrinsic traction forces. ECs also respond to biochemical cues from the surrounding environment by releasing compounds, such as nitric oxide (NO) and Prostaglandins, to tune the vascular tone.^27^ These mechanisms allow the vascular adaptation to muscular activity, ensuring the supply of oxygen and nutrients and the removal of waste by‐products. It is believed that the wide set of environmental stimuli to which ECs are exposed impacts their remodeling and specialization. Different transcriptional programs are then activated defining an arterial (e.g. *FOXC* and *SOXF*)^36^ and venous (e.g. *EPHB4*)^37^ or lymphatic (e.g. *NR2F2*)^38^ EC differentiation. Other transcription factors confer organ‐specific attributes to ECs.^39^, ^40^ Markers characterizing muscle ECs are the genes *E74A*, *ELK1*, *ELK4*, *GABPA*, *NRF‐2, TSPAN7* and the secretion of the angiocrine factor CD36.^41^

Pericytes are the other cell type composing the microvasculature. They have a peri‐endothelial distribution and share with ECs a basement membrane. The fine finger‐like projections characterizing pericyte shape make contact with the underlying and neighboring capillary ECs. The ratio between these two populations changes according to the tissue. In the skeletal muscle, the ratio is very high (100:1 ECs to pericytes), while in the brain it is very low (1:1).^42^

Considering their high number and ubiquitous distribution, ECs represent a relevant cell population to focus on when looking for stromal cells that contribute to the fibrotic process. Indeed, one of the key properties of ECs is their ability to undergo plasticity, which refers to their ability to change and adapt in response to different stimuli.^43^ This plasticity is mediated by a variety of signaling pathways and transcriptional regulators that control gene expression and EC function. For example, ECs can undergo phenotypic changes in response to various types of stresses, such as inflammation,^44^ hypoxia and oxidative stress,^45^ and to mechanical properties (e.g. increase in stiffness observed in fibrotic tissues).^46^ The most important biochemical cues associated with endothelial plasticity are TGFβ,^47^ CTGF,^48^ FGF,^49^ and VEGFA.^50^ A key example of EC plasticity is the endothelial‐hematopoietic transition. During embryogenesis, hematopoietic stem cells develop from the hemogenic endothelium, and their vascular commitment is specified in a Notch‐dependent manner.^51^ Another important example of cellular plasticity is EndoMT, the process that takes part in the embryonal formation of the tricuspid and mitral heart valves, and that gives origin to other cardiac progenitors (e.g. cardiac fibroblasts and smooth muscle cells).^52^

**FIGURE 2:**
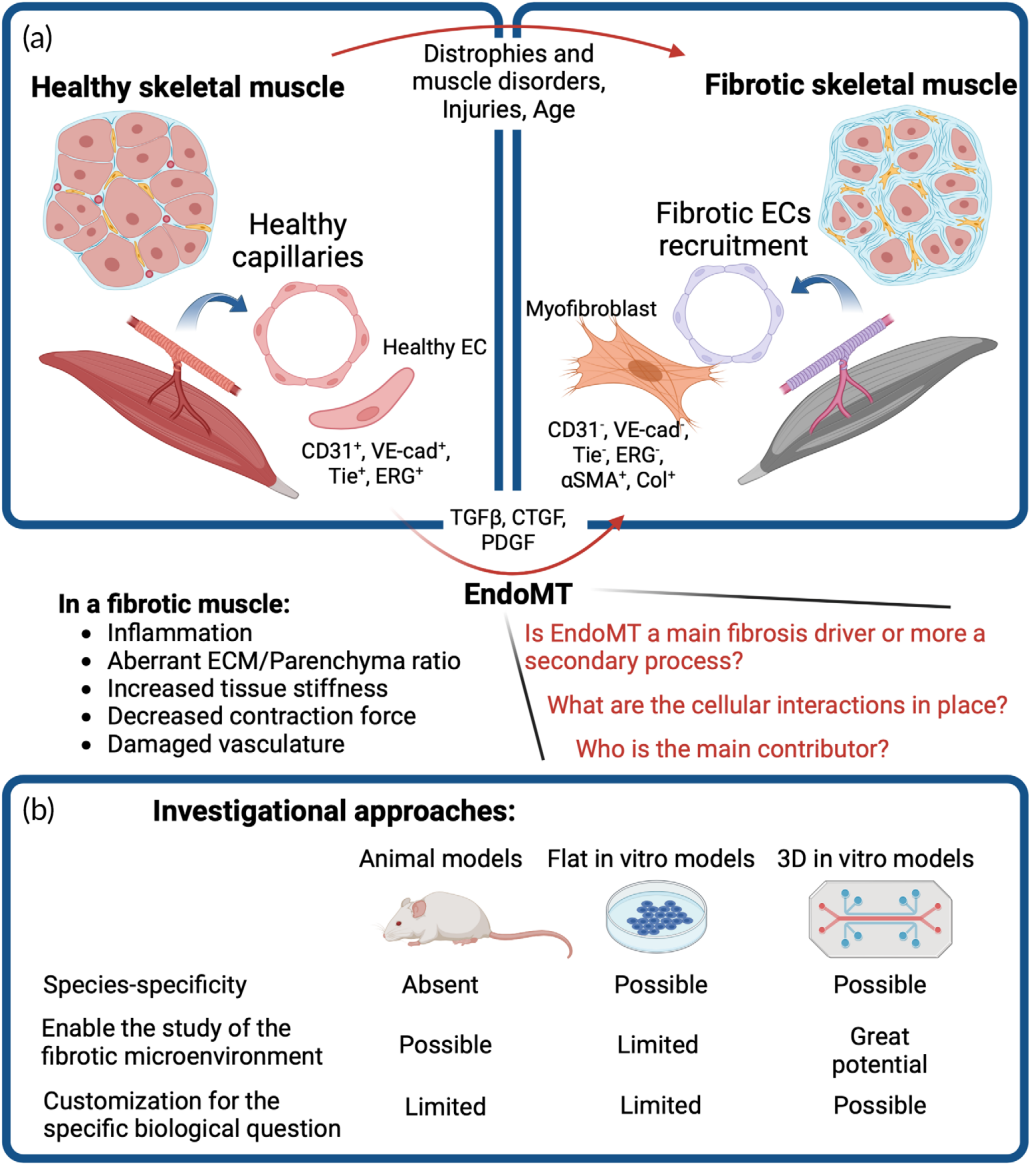
(a) Schematics showing the changes occurring in the skeletal muscle affected by fibrosis and the impact of the disease on endothelial cells (ECs). Summary of the most relevant markers and growth factors related to endothelial‐mesenchymal transition (EndoMT). (b) Available approaches to study EndoMT are animal models and in vitro models. In vitro models can be divided into flat traditional cultures and 3D models. These 3D models, including microphysiological systems, showed huge advancements in recent years and are useful candidates to unveil the pathological mechanisms of cell–cell and cell–matrix communications occurring in fibrotic muscles.

**FIGURE 3:**
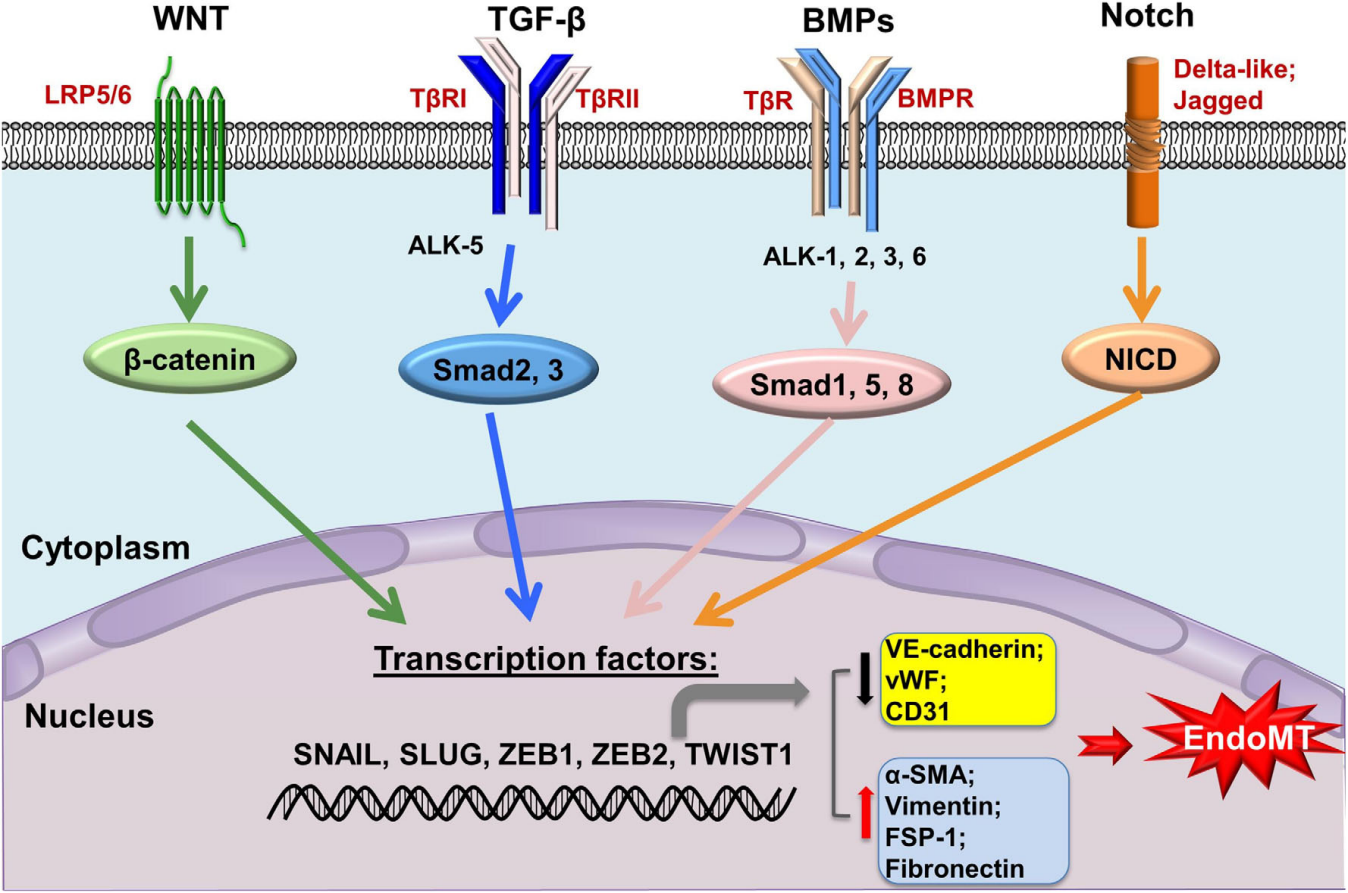
Schematic of the most important signaling pathways involved in the EndoMT initiation, namely TGF‐β, BMP, Wnt/β‐catenin, and Notch. *SNAIL*, *SLUG, ZEB1, ZEB2*, and *TWIST1* are among the transcription factors that participate to the cascade. As a result, the expression of EC markers (such as VE‐cadherin, vWF, and CD31) decreases and the expression of mesenchymal cell markers (like α‐SMA, vimentin, FSP‐1, and fibronectin) increases. Reproduced from Lu et al.^59^

Moreover, it is important to consider that EC plasticity is not limited to the embryonic phase. This concept finds confirmations in the overlapping transcriptome among different cell populations, as shown by single‐cell RNA sequencing studies and fate‐mapping studies.^53^ A recent example is the study by Cameron and colleagues which used single‐cell RNA sequencing to demonstrate the presence of a mixed population of cells co‐expressing pericyte and EC markers in the human skeletal muscle.^54^ While pure ECs and pure pericytes have completely different transcriptional profile, the hybrid population was characterized by the expression of 257 genes in common with pure ECs and 288 genes in common with pure pericytes. These results suggested that a potential transdifferentiation between the two cell populations may be possible. EndoMT drives mature ECs out of their quiescent state^55^, ^56^ to acquire a mesenchymal phenotype characterized by an increased migration capacity and augmented ECM secretion. Morphologically, during EndoMT ECs lose the cell–cell junctions that maintain the barrier properties of the vascular wall and undergo a cytoskeletal reorganization passing from their cobblestone‐like shape to spindle‐like shape without apical‐basal polarity^57^ (Figure 2). From the molecular perspective, EndoMT is generally identified by the reduced expression of CD31/PECAM‐1, Tie1, Tie2, Von‐Willebrand factor, VE‐cadherin, and by the increased expression of myofibroblast markers including alpha‐SMA and FSP‐1.^58^ These changes are directed by four main signaling pathways, initiated by membrane receptors of TGFβ, WNT, BMP and Notch families (Figure 3).^59^ The molecular cascades involve transcription factors such as *TWIST1*, β‐catenin, and *SNAI1*, *2*, *3*, *5*, *8*, as observed in a mouse model of kidney fibrosis.^60^ However, the underneath molecular process is even more convoluted, as new discoveries highlight. For example, the recent in vivo study by Mastej and colleagues showed the disruption of the combinatorial mechanism partnered by *KLF2* and *KLF4*, two transcription factors expressed by quiescent healthy ECs in the context of pulmonary fibrosis.^61^ The decreased expression of *KLF4* drives the upregulation of *KLF2*, which ultimately leads to an EndoMT phenotype. These changes affect the properties of ECs, such as their barrier function and the ability to undergo angiogenesis and vasculogenesis.

EndoMT was associated with inflammation, where combinations of pro‐inflammatory cytokines, such as Angpt2^62^ and Nur77^63^ are necessary to stabilize abnormal vascular remodeling.^44^ Shear stress is also a fundamental factor for vascular homeostasis, and a disturbed flow or low shear stress can promote EndoMT.^64^ In particular, low shear stress can induce EndoMT through a process controlled by the transcription factor *SNAIL*.^65^ Finally, increased ECM stiffness is another potential EndoMT mediator. Another not fully‐deciphered EndoMT promoter is represented by metabolic alterations in ECs, such as the role of fatty acid oxidation.^66^

Given this wide range of promoting conditions, it becomes evident that EC plasticity is important in many physiological and pathological processes, including fibrosis,^30^, ^67^ atherosclerosis^68^ and cancer.^30^ Indeed, EndoMT plays a critical role in maintaining blood vessel homeostasis and in promoting repair and regeneration following injury. However, its dysregulation has been linked to the development of various fibrotic diseases, including cardiac fibrosis.^69^ Eventually, the disruption of the vascular integrity generates leaky capillaries, worsening the chronic inflammation present in fibrotic tissues. Given the similarities in the expression profile of ECs in both the heart and the skeletal muscle,^41^ there is a high likelihood that EndoMT is also involved in skeletal muscle fibrosis. Unfortunately, studies exploring this topic are limited, and there is a significant gap in our understanding of the role of EndoMT in skeletal muscle fibrosis that needs to be filled. In this scenario, 3D in vitro models could provide a major contribution. Microphysiological systems allow the generation of perfusable microvascular networks that replicate the in vivo microvasculature in terms of vessel geometry, ECM composition and dimensionality, cellular architecture and molecular profiles, also in response to biophysical stimuli (e.g. flow).^70^, ^71^ Studies based on this technology observed increased barrier function and junctional reorganization in ECs (such as expression and localization of Occludin) in response to steady and pulsatile shear stress.^72^ Furthermore, microfluidic devices were used to correlate changes in endothelial permeability, sprouting activity, and monolayer integrity following the application of fluid forces and VEGF gradients.^73^, ^74^, ^75^ Additionally, modifying the ECM stiffness it was possible to obtain changes in flow responsiveness and vessel barrier function,^76^, ^77^ as well as in neovessel sprouting, angiogenesis‐associated vessel elongation, and vascular network reorganization.^78^, ^79^

## 4 ADVANCEMENTS OF 3D IN VITRO SKELETAL MUSCLE MODELS

**FIGURE 4:**
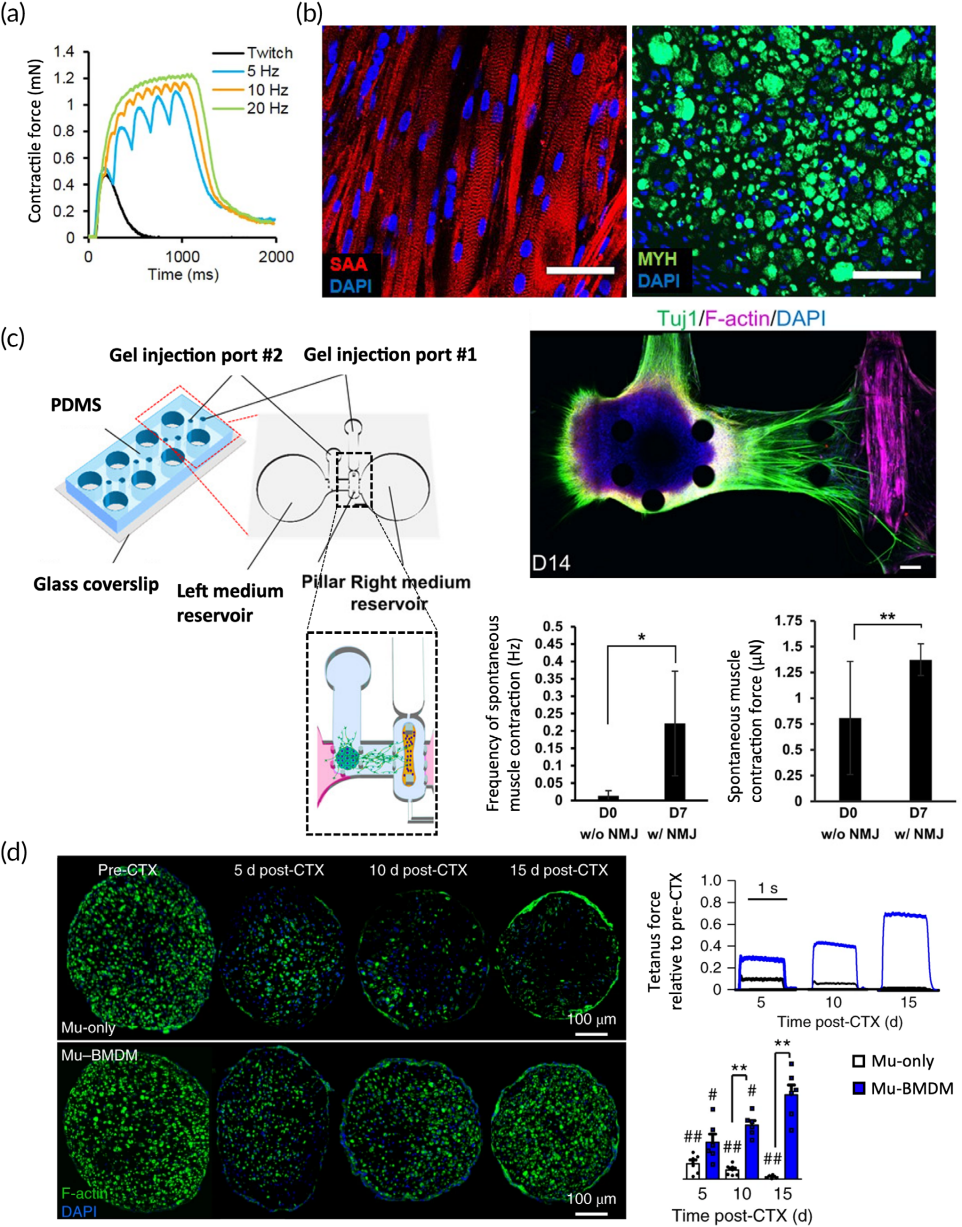
Overview of results achieved by 3D in vitro models of skeletal muscle. (a) Contractile force generated by an engineered 3D human muscle model upon electrical stimulation.^82^ (b) 3D bundle of human differentiated muscle cells (SAA^+^, MYH^+^) observed at different magnifications.^82^ Scale bar = 50 μm. (c) Microfluidic model of a neuromuscular junction (NMJ) showing neural‐induced muscle contraction. A neuronal spheroid (Tuj1^+^) connects to a murine muscle bundle (F‐actin^+^). Scale bar = 100 μm.^83^ (d) 3D model made of a bundle of murine muscle cells (Mu‐only) and muscle cells co‐cultured with macrophages (Mu‐BMDM). The model showed an improved regenerative capacity obtained by the co‐culture after Cardiotoxin‐induced damage.^84^

In recent years, great progress has been achieved in the design of human muscle models (Figure 4a and 4b). Embedding myoblasts in a 3D hydrogel has been identified as the main approach to promote the differentiation of multinucleated myotubes packed together in a bundle.^80^ Gene expression analyses demonstrated that these 3D muscle models were more similar to native muscle tissues compared to 2D cultures.^81^ Interestingly, several muscle‐specific genes were downregulated in 2D cultures compared to 3D models, hence showing that their expression profile was closer to undifferentiated myoblasts. To achieve superior maturation, 3D muscle constructs were also coupled with electrical, optogenetic, or chemical stimulation systems to achieve an active contraction.^85^ Indeed, similarly to in vivo physical exercise, electrical stimulation of 3D in vitro muscles led to a transcriptional increase of *PGC1⍺*, *PDK4*, and other myokines (e.g. *NAMPT* and *ANGPTL4*). Furthermore, electrical stimulation increased the expression of contractile proteins such as myosin heavy chain, improved the synchronicity between calcium transients and contraction, and allowed to recapitulate metabolic effects like GLUT4 translocation and glucose uptake in muscle and cardiac tissues.^86^ In addition, this functional activation allowed to quantify muscle force in different experimental conditions.^87^

Another key feature of 3D muscle models is the presence of side populations of satellite cells, the resident pool of muscular progenitors.^82^ These cells are generally absent in traditional 2D cultures of myoblasts,^88^, ^89^ hence demonstrating an improved capacity of mimicking specific aspects of the native composition and spatial organization of skeletal muscle. In this context, 3D muscle models overcome the limitations of 2D systems offering the chance to study how muscle cells interact with the surrounding microenvironment, namely the ECM and the other cell populations (e.g. fibroblasts, ECs, pericytes). To design physiological ECM, natural hydrogels mainly made of collagen, fibrin, Matrigel, gelatin, or their combinations have been employed. These hydrophilic matrices provide structural support to muscle cells together with molecular interactions that stimulate the maturation of myofibers.^90^ This aspect is of paramount importance to study muscle pathologies where the ECM plays a role, like fibrosis.^91^ Hinds and colleagues tested in a 3D setup various matrix proteins, including collagen I, fibrin, and Matrigel in different concentrations, to develop skeletal muscle bundles based on neonatal rat cells.^92^ The authors compared the impact of these ECM proteins on tissue structure, generation of contractile force, and intracellular Ca^2+^ handling. As a result, a combination of fibrin (4–6 mg/mL) and Matrigel (10%–40%) was able to generate a higher force compared to lower concentrations of the same proteins and to the collagen‐based hydrogels. These results support the use of fibrin and laminin‐rich hydrogels (e.g. Matrigel) for muscle modeling. Moreover, by avoiding the artificial introduction of collagen in the 3D model, it is possible to use its quantification as a fibrotic indicator. The use of artificially manufactured scaffolds offers the great advantage of knowing the biochemical composition and controlling the material stiffness. This versatility retains great value to study the role played by tissue stiffness in fibrosis progression.^93^

Such hydrogel/cell mixtures can be seeded manually or deposited by a bioprinter. The best choice should be made to meet the requirements of the specific application. While bioprinting offers a higher degree of seeding precision, which can be exploited to ensure muscle cell alignment and promote myotube fusion, it also imposes restrictions to the device geometry and hydrogel composition. For further discussion on this matter, we suggest referring to the review by Xiang et al.^94^ As an alternative solution, decellularized muscle tissues were used as 3D naturally derived‐scaffolds for the generation of muscle models. These materials offer the advantage of retaining the native muscle architecture and ultrastructure.^95^ However, challenges on homogeneous and controlled recellularization are posed. Additionally, the composition and biophysical properties of decellularized scaffolds cannot be controlled as finely as with the artificially constructed scaffolds.

Regarding the interaction of muscle cells with other cell populations typical of the muscle microenvironment, multiple microscale, and mesoscale devices have been designed to provide compartmentalization and allow the co‐culture of tissue constructs requiring different maturation time frames. This feature enables to maintain in the same system cells with different culture conditions, facilitating the study of heterotypic communications. For instance, microfluidic models were able to integrate neuromuscular junctions (NMJ) that showed active contraction induced by a neuronal spheroid or motor neurons (Figure 4c).^83^, ^96^ However, so far just a few models have taken advantage of this opportunity. In addition, the impact of skeletal muscle models has been limited by the absence of a stromal component. Myofibers are naturally dispersed in a supporting ECM (i.e. endomysium) where other cell types reside and provide fundamental functions. These cell populations include vascular cells, resident macrophages, and fibroblasts. These stromal cells interact with muscle fibers preserving muscle homeostasis and ensuring muscle adaptation and regeneration (Figure 4d). However, a compromised balance among these cell populations is a typical hallmark of fibrosis. For instance, during muscle injuries, the involvement of macrophages initiates an inflammatory response,^97^ which then proceeds with the recruitment of fibroblasts, FAPs, and other mesenchymal progenitors. During this process, the expression level of multiple cytokines is altered, including molecules known to be involved in muscular fibrosis such as CTGF,^98^ TGFβ,^99^ PDGF,^100^ and VEGF.^101^ Similar phenomena are observable in the tumor microenvironment. In particular, tumor‐associated macrophages, which represent a major part of cancer immune infiltrate, can promote EndoMT and compromise the functionality of blood vessels.^102^ Given that myopathies are associated with inflammatory processes and macrophage infiltration, it is likely that these cells promote EndoMT and fibrosis even in these contexts.

Molecular and cellular imbalance affects the vasculature which changes its tone, molecular profile, and permeability.^103^ Ignoring this complexity limits the impact that 3D in vitro models may have in the identification of critical pathological mechanisms of fibrosis. Therefore, we believe that the next generation of muscle models should focus on including a more complex stromal compartment to fully take advantage of the possibilities offered by these systems to study relevant biological processes.

## 5 3D IN VITRO MODELS ARE ADVANCED SOLUTIONS FOR THE STUDY OF HETEROTYPIC CELL–CELL INTERACTIONS

Despite still being at its infancy, in vitro 3D muscle modeling has already demonstrated a great potential in recreating typical cell interactions occurring in the physiological and pathological muscle microenvironment. Indeed, accumulating evidence indicates a pivotal role of stromal interactions in the pathological onset and progression of muscle fibrosis.^104^ A relevant example of the possibility to in vitro study muscle‐stromal interactions was provided by Juhas and colleagues, who included macrophages in a rat muscle bundle achieving an improved regenerative capacity.^84^ The authors attributed this effect to the capacity of macrophages to support satellite cell proliferation and differentiation to limit myofiber apoptosis and to dampen pro‐inflammatory conditions (Figure 4d). Due to their ubiquitousness and involvement in muscular diseases, ECs have also been a target of tissue engineering, which has been aiming to introduce an organized capillary network in muscle models. The presence of a mutual positive influence between human muscle cells and ECs was confirmed in an in vitro study by the Chazaud lab. By seeding human umbilical vein endothelial cells (HUVECs) on Cytodex beads and then embedding them with myoblasts in a fibrin‐based hydrogel, the group showed that ECs were capable of pro‐myogenic properties by stimulating satellite cell migration, proliferation, and terminal differentiation. In addition, the presence of satellite cells within the gel increased the number of lumenized capillaries. Moreover, capillary elongation and lumenization increased with the maturation of muscle cells.^105^ Apelin, Oncostatin, and Periostin appeared to be three key molecular effectors correlated to myogenesis and angiogenesis, as demonstrated by antibody‐blocking of these three cytokines which resulted in the inhibition of the positive effects of the co‐culture. It is important to notice that the Apelin gene, which is found overexpressed by ECs in the muscle co‐culture, has been recently observed to be downregulated in human pulmonary artery ECs undergoing EndoMT.^106^ It would be interesting to clarify whether Apelin expression is affected by a dystrophic or inflamed muscle to better understand its role in EndoMT.

**FIGURE 5:**
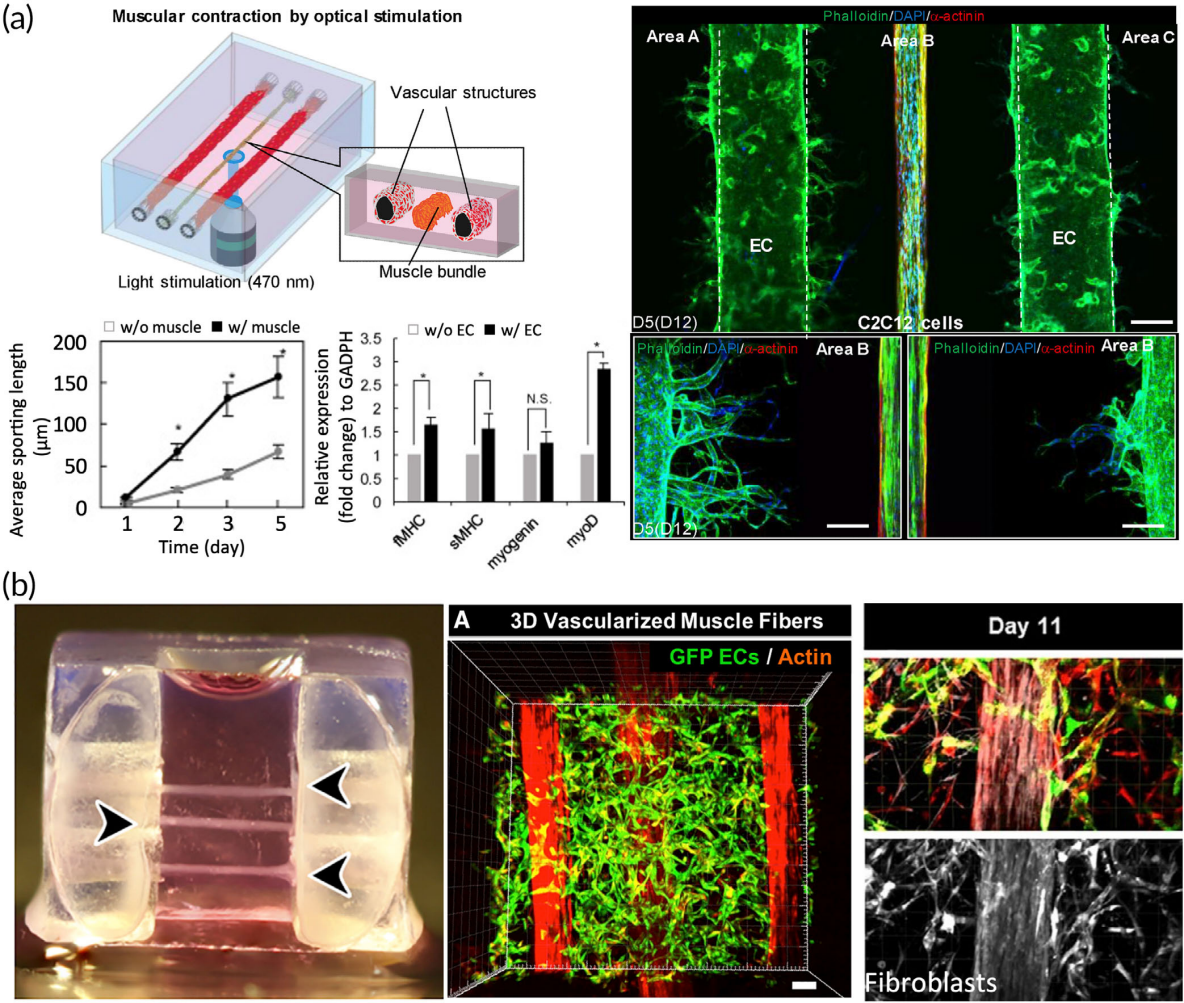
Overview of 3D in vitro models to analyze muscle‐endothelium interactions. (a) Positive mutual cross‐talk between optogenetically engineered mouse skeletal muscles and HUVECs. Scale bar = 100 μm.^108^ (b) 3D vascularized human muscle microenvironment embedding three non‐planar muscle fibers, ECs and muscle fibroblasts to study endothelial plasticity and fibrosis. Scale bar = 100 μm.^109^

In another study, Gholobova and colleagues set up a 3D in vitro co‐culture of human myoblasts and HUVECs.^107^ Myoblasts were seeded in a fibrin hydrogel forming a 3D construct, while HUVECs were added at two different timepoints: contemporarily with the myoblast seeding or after 1 week of myoblast differentiation. The second approach resulted in a better vascular interconnection, lower vascular degradation, and higher myotube formation. While this result demonstrates a positive interaction between differentiated myotubes and HUVECs, the level of myotube maturation obtained was lower when compared to the muscle construct cultured without ECs for the same two‐week interval. This is probably caused by the necessary use of a serum‐rich medium to culture ECs, while myogenesis is promoted by low‐serum medium. Interestingly, Collagen IV, which is overexpressed by microvascular ECs during EndoMT, has been deposited by ECs in these models, proving that matrix protein release could be investigated in these setups. A two‐step seeding approach was also used by Osaki and colleagues. The authors developed a 3D model to demonstrate the mutual cross‐talk between the endothelium and muscle cells (Figure 5a).^108^ First, C2C12/hydrogel mixtures were seeded in a cylindrical cavity created in a sacrificial gelatin template and were left differentiated for 7 days. Then, two parallel endothelialized cylindrical cavities were seeded on both sides of the muscle construct. This work showed that the angiogenesis of HUVECs increased as a result of the co‐culture with C2C12 murine myoblasts. This effect seems to be related to the paracrine bi‐directional signaling based on Neuregulin‐1 (NRG‐1) secreted by ECs and Angiopoietin‐1 production by muscle cells in 5 days of co‐culture. The study revealed an increase in Tie2 expression prompted by muscle co‐culture. This gene is typically downregulated during EndoMT. Tie2 is a receptor tyrosine kinase that has been used for lineage‐tracing studies to demonstrate the involvement of ECs in renal fibrosis.^110^, ^111^ It has also been shown to have anti‐inflammatory effects, suppressing VEGF and TNF expression, which are also targeted by the two drugs currently approved for the treatment of pulmonary fibrosis, Nintedanib and Pirfenidone.^112^ Investigating the effects of muscle co‐culture on Tie2 expression, possibly comparing healthy and dystrophic muscle constructs, or increasing Tie2 expression could be a promising approach to identifying new therapeutic strategies. Indeed, clarifying the recruitment of ECs during fibrosis is essential in finding a way to contrast pro‐fibrotic stimuli. In a different setup, Kim and colleagues showed that starting the co‐culture of HUVECs and human myoblasts after 2 days from the muscle seeding allowed to obtain a greater muscle contraction force, myotube fusion, and vascular network development than with the seeding of HUVECs performed at day 5.^113^ While these results seem to be contradictory with the previous study, it should be considered that the design is different. Indeed, two concentric cylinders were created seeding muscle cells on the outer side and HUVECs in the inner one. Taking advantage of this configuration, the authors were able to flow endothelial basal medium in the inner cylinder (the one in contact with ECs), while muscle differentiation medium was provided on the external environment, surrounding the construct. This solution allowed to provide the appropriate medium to muscle cells and ECs at the same time.

Taken together, these results demonstrate the mutual positive effects of the co‐culture of muscle cells and ECs on myogenesis and angiogenesis. Additionally, it has been proven that cytokines and growth factors involved in EndoMT are impacted by 3D co‐culture setups. This suggests that such tools might be the ideal solution to decipher how the fibrotic recruitment of ECs takes place in diseased muscles.

The possibility to customize the architecture of the system is another relevant characteristic of 3D in vitro models. This architecture can be adapted to perform specific observations and to arrange cell populations in particular settings. This feature can be especially exploited to culture more than one cell population. A successful example is the concentric design used by the Asada group to develop a tri‐culture in vitro model including human myotubes, ECs, and fibroblasts.^114^ Using a system of coaxial needles, the authors were able to culture three concentric cylinders formed by human myoblasts (outer layer), fibroblasts (middle layer), and ECs (inner layer). In agreement with previous studies, the authors confirmed an increased myotube thickness and contractile force promoted by the co‐culture. Moreover, it was demonstrated that the muscle functionality improved according to the distribution of fibroblasts. Indeed, when fibroblasts were localized in the inter‐layer between muscle cells and ECs, a better vascular sprouting and a higher contractile force were registered in comparison to the general distribution of fibroblasts in the same layer occupied by muscle cells. This result demonstrates the importance of spatial cell distribution when studying cellular crosstalk, an aspect majorly exploitable thanks to the compartmentalization allowed by 3D in vitro models. For example, it might be relevant to try to identify a spatial pattern in the fibrotic recruitment of ECs in relation with their proximity to another cell population of the muscle microenvironment.

Overall, these models represent all the successful attempts achieved so far of implementing a stromal muscle compartment together with muscle fibers/bundles (Table 1). These models successfully showed how this approach has provided significant insights to understand cell–cell interactions within muscle tissue. Despite their success, these models have not yet been used to study the pathological evolution of the vasculature in muscular diseases, such as dystrophies. As previously discussed, ECs play a crucial role in tissue maturation and maintenance of homeostasis, making muscle models with ECs ideal for understanding their role in different pathological conditions, including inflammation or fibrosis. Moreover, an additional progress would derive from taking into account the tissue‐specificity of ECs. Indeed, ECs from different tissues differ in terms of transcriptome, proteome, metabolic profile, and functionality.^115^, ^116^ The EC tissue‐specificity might be relevant when studying certain diseases and could also increase the reliability of in vitro models. Unfortunately, very few studies have dealt with EC tissue‐specificity and plasticity, even though some examples have recently paved the way to implement this aspect within 3D in vitro models. The adaptation capacity of ECs was shown in a 3D triculture model of human muscle with ECs and fibroblasts (Figure 5b), whereby ECs acquired muscular phenotypic signatures when co‐cultured with muscle cells.^109^, ^117^ Since changes in EC migration are expected with the mesenchymal transition, it would be interesting to investigate them in a similar setup when a pro‐fibrotic context is created. Another work focused on endothelial tissue specificity, although in a different context, was performed by Osaki and colleagues. The authors supplemented ECs with retinoic acid to obtain an endothelial barrier more similar to the blood brain barrier, useful to study drug penetration.^96^ As far as we know, the usage of 3D muscle models to investigate EndoMT has not yet been exploited, while a few successful works focused on EndoMT in other tissues. Kramer and co‐workers developed a microfluidic‐based system embedding human microvascular endothelial cells to investigate if EndoMT exerts a role since the early stages of systemic sclerosis before the establishment of full‐blown fibrosis.^118^, ^119^ The group observed vascular alterations upon exposure to pro‐inflammatory and pro‐fibrotic cytokines, such as TNFα and TGFβ, mimicking pathological environments. Also, the negative effects of the two cytokines were successfully inhibited via the addition of TNFα and TGFβ inhibitors showing the potential of the model as a drug‐testing tool. Another recent work by Whiteford and colleagues reproduced EndoMT in a liver‐on‐a‐chip.^120^ The group co‐cultured liver sinusoidal endothelial cells and hepatocytes in a microfluidic chip and observed an increased migratory behavior of ECs in a fibrotic context. Finally, Bramsen and colleagues demonstrated the EndoMT‐promoting effect that glycosaminoglycans have on porcine aortic valve ECs in a 3D setup.^121^

**TABLE 1:** Summary of the most advanced 3D in vitro models of skeletal muscle integrating ECs.

Authors	Cell populations	Device	Outcomes
Juhas et al. (2018)	Rat myoblasts Bone marrow‐derived macrophages	3D fibrin and Matrigel‐based cylindrical hydrogel with aligned myobundles and interspersed macrophages	Increased muscle regenerative capacities following cell damage
Latroche et al. (2017)	Human primary myoblasts Human Umbilical Vein Endothelial Cells (HUVECs)	HUVECs seeded on Cytodex beads and cultured with myoblasts on a fibrin‐based hydrogel	The co‐culture improved myogenesis and angiogenesis mainly attributed to a signaling based on Apelin, Oncostatin and Periostin.
Gholobova et al. (2020)	Human primary myoblasts Human Umbilical Vein Endothelial Cells (HUVECs)	Fibrin‐based cylindrical hydrogel with aligned myobundles. HUVECs seeded interspersed in the bundle or around it	The muscle maturation was superior in the two‐step seeding approach, where HUVECs were seeded after myotube differentiation
Osaki et al. (2018)	C2C12 murine myoblasts Human Umbilical Vein Endothelial Cells (HUVECs)	Fibrin‐based bundle of C2C12 created in close proximity of two HUVEC‐endothelialized cylindrical cavities	Both muscle differentiation and HUVEC angiogenesis benefited from the co‐culture, in a paracrine looping‐signaling based on Neuregulin‐1 and Angiopoietin‐1
Bersini et al. (2018)	Human primary myoblasts Human endothelial cells Human primary muscle fibroblasts Human bone‐marrow derived mesenchymal stem cells	Microscale cylindrical muscle bundles surrounded by a fibrin‐based hydrogel with ECs, fibroblasts and mesenchymal cells	Muscle‐specific phenotype of ECs was identified (expression of TSPAN7, PPARG). Recruitment of fibroblasts regulated by paracrine signaling
Kim et al. (2022)	Human primary myoblasts Human Umbilical Vein Endothelial Cells (HUVECs)	Two concentrical layers with myoblasts in the outer side and HUVECs in the inner one	Longer co‐culture improved muscle contraction, fusion, and vascular sprouting
Kim et al. (2022)	Human primary myoblasts Human Umbilical Vein Endothelial Cells (HUVECs) Human primary fibroblasts	Three concentrical layers with myoblasts in the outer side, fibroblasts in the middle layer and HUVECs in the inner one	Co‐culture improved muscle functionality and differentiation. Fibroblast spatial localization impacted muscle functionality

*Note*: These models demonstrated their efficacy to evaluate the beneficial mutual interaction between different healthy cell types.

Despite the importance of EndoMT in the development of fibrosis in various muscular pathologies, there is currently a lack of skeletal muscle 3D in vitro models that can replicate this process. However, given the promising results achieved in other tissues through the study of EndoMT, we believe that the time has come to develop new 3D muscle models that can help us to better understand the mechanisms behind EndoMT during fibrosis.

## 6 CONCLUSIONS AND PERSPECTIVES

Over the last decade, relevant progress has been made in setting up advanced 3D models of human skeletal muscle. The convergence of biological and engineering technologies allows to culture human muscle cells (either induced pluripotent stem cell‐derived^88^ or primary cells) to obtain aligned bundles of differentiated muscle fibers, showing sarcomeric organization and ability to contract upon chemical and electrical stimulation. While the culture conditions to properly expand and mature primary myoblasts have been defined, an aspect that should be considered is the dimension of the model. Successful results have been achieved with both micro^122^ and meso‐scale models,^123^ however the design should be carefully defined according to the requirements of the final analyses, such as number of cells and accessibility of the seeded chamber for cell retrieval.

The EC source is another important factor to take into consideration when developing an in vitro muscle model. HUVECs have been widely used for their high proliferation and their capacity of forming vessel‐like structures. However, their relevance can be questioned. Primary ECs derived from the tissue object of the study could be a more representative cell choice than umbilical vein ECs. Indeed, ECs can be differentiated by tissue‐specific gene expression profiles. In the context of skeletal muscle ECs, this was demonstrated using an in vitro 3D construct embedding primary human muscle fibers and primary human ECs. ECs acquired a muscle‐specific expression of specific genes, such as *TSPAN7* and *PPARG*. Furthermore, the presence of ECs upregulated the expression of Desmin in the myofibers.^109^

To further increase the physiological relevance, multiple cellular populations can be introduced and co‐cultured in the same system. Several co‐culture studies demonstrated the feasibility and potential of multicellular models with ECs,^109^ fibroblasts,^113^ macrophages,^84^ and neurons.^124^ 3D models based on microfluidic principles are the best option to create the compartmentalization of different culture media and, at the same time, to allow cellular crosstalk and migration. The few studies that have integrated the vascular component in muscle in vitro 3D systems successfully observed the positive mutual influence between the two cell populations, improving myogenesis and angiogenesis. In this context, we think that 3D in vitro muscle models could be exploited to study EndoMT, one of the key processes contributing to fibrosis. To our knowledge, no study has addressed the role of EndoMT on human muscular fibrosis, despite it has been demonstrated to be relevant in the pathogenesis of cardiac, pulmonary, cerebral, and renal fibrosis.^30^, ^111^, ^125^, ^126^, ^127^ Many aspects related to this process are still not clear and, most importantly, it is not known how to stop or revert it. For example, it is still unknown how EndoMT relates with muscle tissue inflammation and how other cell populations of the fibrotic microenvironment influence it. A study on murine models showed the presence of EndoMT in skeletal muscle fibrosis and the importance of M1 macrophages for neo‐angiogenesis in muscle regeneration.^128^, ^129^ However, the authors associated this effect to resident vascular progenitors and not to differentiated ECs.^130^ This consideration might explain the apparent contradiction with another study based on murine models where M1 macrophages were identified as promoters of cardiac fibrosis by inducing EndoMT via a SMAD2‐dependent paracrine communication.^131^ Introducing human ECs and macrophages into a 3D in vitro model, along with differentiated myotubes, represents a promising approach to elucidate the relationship between endothelium and macrophages, one of the main infiltrating cells. By comparing the effects of M1 and M2 macrophages, this model could help to clarify whether ECs are primarily affected during the early inflammatory response or the later stages of regeneration. The same approach could also be used to investigate the interactions between ECs and other cell types, such as FAPs and pericytes. Given the presence of multiple cell populations, single‐cell RNA sequencing may be the most suitable analytical method, as it enables the effective identification of transitioning phenotypes, such as ECs undergoing EndoMT. Taking advantage of microfluidic principles, 3D models also offer the opportunity to analyze the perfusability of the vascular network, which can serve as a functional readout of the EC state. Moreover, these models allow to tune the flow rate and shear stress within the vascular networks. This feature would allow us to study the impact of shear stress on EndoMT, since abnormally low shear stress values were shown to promote EndoMT, although the underlying mechanisms are not fully understood. Finally, molecular compounds can be screened to assess whether they exert a therapeutic effect preventing or reverting EndoMT. Currently, Nintedanib^132^ and Pirfenidone^133^ are the only two drugs approved for the specific treatment of idiopathic pulmonary fibrosis. Since these drugs act on a wide spectrum of growth factor receptors that are associated with EndoMT, it would be particularly interesting to investigate if they have a beneficial effect on vascularized muscle models showing signs of this mesenchymal transition.

In this review, the most relevant solutions to model in vitro 3D vascularized skeletal muscle were reported. There is no exclusive best design, these systems should be optimized according to the specific experimental requirements. Indeed, the versatility of these systems is one of the most important benefits they bring when studying a specific biological question.

In conclusion, 3D models offer several advantages over traditional 2D cultures, including the ability to mimic the complex cell–cell and cell–matrix interactions observed in vivo. These models also allow for the integration of multiple cell types and the incorporation of mechanical and biochemical cues that can influence cell behavior. We believe that this approach is particularly promising to investigate pathological mechanisms like EndoMT, which might be the result of different contributing factors. These advanced models offer superior experimental possibilities like multicellular cultures and functional readouts, improving the relevance of in vitro drug discovery. Hence, 3D muscle models could greatly accelerate the identification and development of effective therapeutics targeting EndoMT and tissue fibrosis.

## AUTHOR CONTRIBUTIONS

**Riccardo Francescato:** Conceptualization (equal); visualization (lead); writing – original draft (lead); writing – review and editing (equal). **Matteo Moretti:** Funding acquisition (equal); supervision (equal); writing – review and editing (equal). **Simone Bersini:** Conceptualization (equal); funding acquisition (equal); supervision (equal); writing – review and editing (equal).

## FUNDING INFORMATION

The project has received funding from the European Union's Horizon 2020 research and innovation programme under the Marie Skłodowska‐Curie grant agreement No 860715. This work was supported by the Swiss State Secretariat for Education, Research and Innovation (SERI) under contract number MB22.00085. Simone Bersini greatly acknowledges Fondation Suisse de Recherche sur les Maladies Musculaires and Novartis Foundation for medical‐biological research.

## CONFLICT OF INTEREST STATEMENT

The authors declare no conflict of interest.

## Data Availability

Data sharing is not applicable to this article as no new data were created or analyzed in this study.

## References

[1] Rockey DC , Bell PD , Hill JA . Fibrosis—a common pathway to organ injury and failure. New Engl J Med. 2015;372:1138‐1149. 25785971 10.1056/NEJMra1300575

[2] Richeldi L , du Bois RM , Raghu G , et al. Efficacy and safety of nintedanib in idiopathic pulmonary fibrosis. New Engl J Med. 2014;370:2071‐2082. 24836310 10.1056/NEJMoa1402584

[3] Taniguchi H , Ebina M , Kondoh Y , et al. Pirfenidone in idiopathic pulmonary fibrosis. Eur Respir J. 2010;35:821‐829. 19996196 10.1183/09031936.00005209

[4] Desguerre I , Mayer M , Leturcq F , Barbet JP , Gherardi RK , Christov C . Endomysial fibrosis in Duchenne muscular dystrophy: a marker of poor outcome associated with macrophage alternative activation. J Neuropathol Exp Neurol. 2009;68:762‐773. 19535995 10.1097/NEN.0b013e3181aa31c2

[5] Schakman O , Kalista S , Barbé C , Loumaye A , Thissen JP . Glucocorticoid‐induced skeletal muscle atrophy. Int J Biochem Cell Biol. 2013;45:2163‐2172. 23806868 10.1016/j.biocel.2013.05.036

[6] Mahdy MAA . Skeletal muscle fibrosis: an overview. Cell Tissue Res. 2018;375(3):575‐588. 30421315 10.1007/s00441-018-2955-2

[7] Corona BT , Wenke JC , Ward CL . Pathophysiology of volumetric muscle loss injury. Cells Tissues Organs. 2016;202:180‐188. 27825160 10.1159/000443925

[8] Theadom A , Rodrigues M , Roxburgh R , et al. Prevalence of muscular dystrophies: a systematic literature review. Neuroepidemiology. 2015;43:259‐268. 10.1159/000369343 25532075

[9] Grasman JM , Zayas MJ , Page RL , Pins GD . Biomimetic scaffolds for regeneration of volumetric muscle loss in skeletal muscle injuries. Acta Biomater. 2015;25:2‐15. 26219862 10.1016/j.actbio.2015.07.038 PMC4562809

[10] Bersini S , Gilardi M , Mora M , et al. Tackling muscle fibrosis: from molecular mechanisms to next generation engineered models to predict drug delivery. Adv Drug Deliv Rev. 2018;129:64‐77. 29518415 10.1016/j.addr.2018.02.009

[11] Moysidou CM , Barberio C , Owens RM . Advances in engineering human tissue models. Front Bioeng Biotechnol. 2020;8:620962. 33585419 10.3389/fbioe.2020.620962 PMC7877542

[12] Vunjak‐Novakovic G , Ronaldson‐Bouchard K , Radisic M . Organs‐on‐a‐chip models for biological research. Cell. 2021;184:4597‐4611. 34478657 10.1016/j.cell.2021.08.005 PMC8417425

[13] Bersini S , Arrigoni C , Lopa S , Bongio M , Martin I , Moretti M . Engineered miniaturized models of musculoskeletal diseases. Drug Discov Today. 2016;21:1429‐1436. 27132520 10.1016/j.drudis.2016.04.015

[14] Piera‐Velazquez S , Jimenez SA . Endothelial to mesenchymal transition: role in physiology and in the pathogenesis of human diseases. Physiol Rev. 2019;99:1281‐1324. 30864875 10.1152/physrev.00021.2018 PMC6734087

[15] Ismaeel A , Kim JS , Kirk JS , Smith RS , Bohannon WT , Koutakis P . Role of transforming growth factor‐β in skeletal muscle fibrosis: a review. Int J Mol Sci. 2019;20:2446. 31108916 10.3390/ijms20102446 PMC6566291

[16] Meng XM , Nikolic‐Paterson DJ , Lan HY . TGF‐β: the master regulator of fibrosis. Nat Rev Nephrol. 2016;12:325‐338. 27108839 10.1038/nrneph.2016.48

[17] Tschumperlin DJ , Ligresti G , Hilscher MB , Shah VH . Mechanosensing and fibrosis. J Clin Invest. 2018;128:74‐84. 29293092 10.1172/JCI93561 PMC5749510

[18] Lampi MC , Reinhart‐King CA . Targeting extracellular matrix stiffness to attenuate disease: from molecular mechanisms to clinical trials. Sci Transl Med. 2018;10:475. 10.1126/scitranslmed.aao0475 29298864

[19] Heo SJ , Thorpe SD , Driscoll TP , Duncan RL , Lee DA , Mauck RL . Biophysical regulation of chromatin architecture instills a mechanical memory in mesenchymal stem cells. Sci Rep. 2015;5:16895. 26592929 10.1038/srep16895 PMC4655352

[20] Mack M . Inflammation and fibrosis. Matrix Biol. 2018;68–69:106‐121. 10.1016/j.matbio.2017.11.010 29196207

[21] Wilson SE , Chaurasia SS , Medeiros FW . Apoptosis in the initiation, modulation and termination of the corneal wound healing response. Exp Eye Res. 2007;85:305‐311. 17655845 10.1016/j.exer.2007.06.009 PMC2039895

[22] Gillies AR , Lieber RL . Structure and function of the skeletal muscle extracellular matrix. Muscle Nerve. 2011;44:318‐331. 21949456 10.1002/mus.22094 PMC3177172

[23] Tabib T , Huang M , Morse N , et al. Myofibroblast transcriptome indicates SFRP2hi fibroblast progenitors in systemic sclerosis skin. Nat Commun. 2021;12(1):4384. 34282151 10.1038/s41467-021-24607-6 PMC8289865

[24] Hinz B . Myofibroblasts. Exp Eye Res. 2016;142:56‐70. 26192991 10.1016/j.exer.2015.07.009

[25] Pessina P , Kharraz Y , Jardí M , et al. Fibrogenic cell plasticity blunts tissue regeneration and aggravates muscular dystrophy. Stem Cell Rep. 2015;4:1046‐1060. 10.1016/j.stemcr.2015.04.007 PMC4472037 25981413

[26] Dulauroy S , di Carlo SE , Langa F , Eberl G , Peduto L . Lineage tracing and genetic ablation of ADAM12+ perivascular cells identify a major source of profibrotic cells during acute tissue injury. Nat Med. 2012;18(8):1262‐1270. 22842476 10.1038/nm.2848

[27] Uezumi A , Ito T , Morikawa D , et al. Fibrosis and adipogenesis originate from a common mesenchymal progenitor in skeletal muscle. J Cell Sci. 2011;124:3654‐3664. 22045730 10.1242/jcs.086629

[28] Madaro L , Passafaro M , Sala D , et al. Denervation‐activated STAT3–IL‐6 signalling in fibro‐adipogenic progenitors promotes myofibres atrophy and fibrosis. Nat Cell Biol. 2018;20(8):917‐927. 30050118 10.1038/s41556-018-0151-y PMC6145844

[29] Leinroth AP , Mirando AJ , Rouse D , et al. Identification of distinct non‐myogenic skeletal‐muscle‐resident mesenchymal cell populations. Cell Rep. 2022;39:110785. 35545045 10.1016/j.celrep.2022.110785 PMC9535675

[30] Zeisberg EM , Tarnavski O , Zeisberg M , et al. Endothelial‐to‐mesenchymal transition contributes to cardiac fibrosis. Nat Med. 2007;13:952‐961. 17660828 10.1038/nm1613

[31] Dort J , Fabre P , Molina T , Dumont NA . Macrophages are key regulators of stem cells during skeletal muscle regeneration and diseases. Stem Cells Int. 2019;2019:1‐20. 10.1155/2019/4761427 PMC6664695 31396285

[32] Xiao L , Dudley AC . Fine‐tuning vascular fate during endothelial–mesenchymal transition. J Pathol. 2017;241:25‐35. 27701751 10.1002/path.4814 PMC5164846

[33] Piera‐Velazquez S , Li Z , Jimenez SA . Role of endothelial‐mesenchymal transition (EndoMT) in the pathogenesis of fibrotic disorders. Am J Pathol. 2011;179:1074‐1080. 21763673 10.1016/j.ajpath.2011.06.001 PMC3157273

[34] Frontera WR , Ochala J . Skeletal muscle: a brief review of structure and function. Behav Genet. 2015;45:183‐195. 10.1007/s00223-014-9915-y 25294644

[35] Yin H , Arpino JM , Lee JJ , Pickering JG . Regenerated microvascular networks in ischemic skeletal muscle. Front Physiol. 2021;12:662073. 34177614 10.3389/fphys.2021.662073 PMC8231913

[36] Corada M , Morini MF , Dejana E . Signaling pathways in the specification of arteries and veins. Arterioscler Thromb Vasc Biol. 2014;34:2372‐2377. 25169934 10.1161/ATVBAHA.114.303218

[37] Wolf K , Hu H , Isaji T , Dardik A . Molecular identity of arteries, veins, and lymphatics. J Vasc Surg. 2019;69:253‐262. 30154011 10.1016/j.jvs.2018.06.195 PMC6309638

[38] Marcelo KL , Goldie LC , Hirschi KK . Regulation of endothelial cell differentiation and specification. Circ Res. 2013;112:1272‐1287. 23620236 10.1161/CIRCRESAHA.113.300506 PMC3768127

[39] Potente M , Mäkinen T . Vascular heterogeneity and specialization in development and disease. Nat Rev Mol Cell Biol. 2017;18:477‐494. 28537573 10.1038/nrm.2017.36

[40] Koch PS , Lee KH , Goerdt S , Augustin HG . Angiodiversity and organotypic functions of sinusoidal endothelial cells. Angiogenesis. 2021;24(2):289‐310. 33745018 10.1007/s10456-021-09780-y PMC7982081

[41] Nolan DJ , Ginsberg M , Israely E , et al. Molecular signatures of tissue‐specific microvascular endothelial cell heterogeneity in organ maintenance and regeneration. Dev Cell. 2013;26:204‐219. 23871589 10.1016/j.devcel.2013.06.017 PMC3873200

[42] Shepro D , Morel NML . Pericyte physiology. FASEB J. 1993;7:1031‐1038. 8370472 10.1096/fasebj.7.11.8370472

[43] Greenspan LJ , Weinstein BM . To be or not to be: endothelial cell plasticity in development, repair, and disease. Angiogenesis. 2021;24(2):251‐269. 33449300 10.1007/s10456-020-09761-7 PMC8205957

[44] Yoshimatsu Y , Watabe T . Emerging roles of inflammation‐mediated endothelial–mesenchymal transition in health and disease. Inflamm Regen. 2022;42:1‐21. 35130955 10.1186/s41232-021-00186-3 PMC8818500

[45] Thuan DTB , Zayed H , Eid AH , et al. A potential link between oxidative stress and endothelial‐to‐mesenchymal transition in systemic sclerosis. Front Immunol. 2018;9:385511. 10.3389/fimmu.2018.01985 PMC6156139 30283435

[46] ten Dijke P , Egorova AD , Goumans MJTH , Poelmann RE , Hierck BP . TGF‐β signaling in endothelial‐to‐mesenchymal transition: the role of shear stress and primary cilia. Sci Signal. 2012;5(212):pt2. 10.1126/scisignal.2002722 22355187

[47] Dejana E , Hirschi KK , Simons M . The molecular basis of endothelial cell plasticity. Nat Commun. 2017;8:14361. 28181491 10.1038/ncomms14361 PMC5309780

[48] He M , Chen Z , Martin M , et al. MiR‐483 targeting of CTGF suppresses endothelial‐to‐mesenchymal transition: therapeutic implications in Kawasaki disease. Circ Res. 2017;120:354‐365. 27923814 10.1161/CIRCRESAHA.116.310233 PMC5391835

[49] de Smet F , Tembuyser B , Lenard A , et al. Fibroblast growth factor signaling affects vascular outgrowth and is required for the maintenance of blood vessel integrity. Chem Biol. 2014;21:1310‐1317. 25200605 10.1016/j.chembiol.2014.07.018

[50] Li X , Padhan N , Sjöström EO , et al. VEGFR2 pY949 signalling regulates adherens junction integrity and metastatic spread. Nat Commun. 2016;7:11017. 27005951 10.1038/ncomms11017 PMC4814575

[51] Ditadi A , Sturgeon CM , Tober J , et al. Human definitive haemogenic endothelium and arterial vascular endothelium represent distinct lineages. Nat Cell Biol. 2015;17(5):580‐591. 25915127 10.1038/ncb3161 PMC4551438

[52] Gritz E , Hirschi KK . Specification and function of hemogenic endothelium during embryogenesis. Cellular and Molecular Life Sciences. 2016;73(8):1547‐1567. 26849156 10.1007/s00018-016-2134-0 PMC4805691

[53] Pasut A , Becker LM , Cuypers A , Carmeliet P . Endothelial cell plasticity at the single‐cell level. Angiogenesis. 2021;24(2):311‐326. 34061284 10.1007/s10456-021-09797-3 PMC8169404

[54] Cameron A , Wakelin G , Gaulton N , et al. Identification of underexplored mesenchymal and vascular‐related cell populations in human skeletal muscle. Am J Physiol Cell Physiol. 2022;323:C1586‐C1600. 36342160 10.1152/ajpcell.00364.2022

[55] Schlereth K , Weichenhan D , Bauer T , et al. The transcriptomic and epigenetic map of vascular quiescence in the continuous lung endothelium. Elife. 2018;7:e34423. 10.7554/eLife.34423 PMC5947988 29749927

[56] Ricard N , Bailly S , Guignabert C , Simons M . The quiescent endothelium: signalling pathways regulating organ‐specific endothelial normalcy. Nat Rev Cardiol. 2021;18(8):565‐580. 33627876 10.1038/s41569-021-00517-4 PMC7903932

[57] Sanchez‐Duffhues G , Orlova V , ten Dijke P . In brief: endothelial‐to‐mesenchymal transition. J Pathol. 2016;238:378‐380. 26446982 10.1002/path.4653

[58] Lovisa S , Fletcher‐Sananikone E , Sugimoto H , et al. Endothelial‐to‐mesenchymal transition compromises vascular integrity to induce Myc‐mediated metabolic reprogramming in kidney fibrosis. Sci Signal. 2020;13:eaaz2597. 32518142 10.1126/scisignal.aaz2597 PMC7790440

[59] Lu X , Gong J , Dennery PA , Yao H . Endothelial‐to‐mesenchymal transition: pathogenesis and therapeutic targets for chronic pulmonary and vascular diseases. Biochem Pharmacol. 2019;168:100‐107. 31251941 10.1016/j.bcp.2019.06.021 PMC6733623

[60] Platel V , Faure S , Corre I , Clere N . Endothelial‐to‐mesenchymal transition (EndoMT): roles in tumorigenesis, metastatic extravasation and therapy resistance. J Oncol. 2019;2019:1‐13. 10.1155/2019/8361945 PMC6701373 31467544

[61] Mastej V , Axen C , Wary A , Minshall RD , Wary KK . A requirement for Krüppel like Factor‐4 in the maintenance of endothelial cell quiescence. Front Cell Dev Biol. 2022;10:1003028. 36425528 10.3389/fcell.2022.1003028 PMC9679496

[62] Lee SJ , Lee CK , Kang S , et al. Angiopoietin‐2 exacerbates cardiac hypoxia and inflammation after myocardial infarction. J Clin Invest. 2018;128:5018‐5033. 30295643 10.1172/JCI99659 PMC6205384

[63] Chen J , Jia J , Ma L , et al. Nur77 deficiency exacerbates cardiac fibrosis after myocardial infarction by promoting endothelial‐to‐mesenchymal transition. J Cell Physiol. 2021;236:495‐506. 32542822 10.1002/jcp.29877

[64] Afshar Y , Ma F , Quach A , et al. Transcriptional drifts associated with environmental changes in endothelial cells. Elife. 2023;12:e81370. 10.7554/eLife.81370 PMC10168696 36971339

[65] Mahmoud MM , Serbanovic‐Canic J , Feng S , et al. Shear stress induces endothelial‐to‐mesenchymal transition via the transcription factor Snail. Sci Rep. 2017;7:3375. 28611395 10.1038/s41598-017-03532-z PMC5469771

[66] Xiong J , Kawagishi H , Yan Y , et al. A metabolic basis for endothelial‐to‐mesenchymal transition. Mol Cell. 2018;69:689‐698.e7. 29429925 10.1016/j.molcel.2018.01.010 PMC5816688

[67] He J , Xu Y , Koya D , Kanasaki K . Role of the endothelial‐to‐mesenchymal transition in renal fibrosis of chronic kidney disease. Clin Exp Nephrol. 2013;17:488‐497. 23430391 10.1007/s10157-013-0781-0

[68] Chen PY , Qin L , Baeyens N , et al. Endothelial‐to‐mesenchymal transition drives atherosclerosis progression. J Clin Invest. 2015;125:4514‐4528. 26517696 10.1172/JCI82719 PMC4665771

[69] Islam S , Boström KI , di Carlo D , et al. The mechanobiology of endothelial‐to‐mesenchymal transition in cardiovascular disease. Front Physiol. 2021;12:734215. 34566697 10.3389/fphys.2021.734215 PMC8458763

[70] Kutys ML , Chen CS . Forces and mechanotransduction in 3D vascular biology. Curr Opin Cell Biol. 2016;42:73‐79. 27209346 10.1016/j.ceb.2016.04.011 PMC5064809

[71] Majewska A , Wilkus K , Brodaczewska K , Kieda C . Endothelial cells as tools to model tissue microenvironment in hypoxia‐dependent pathologies. Int J Mol Sci. 2021;22:520. 33430201 10.3390/ijms22020520 PMC7825710

[72] Price GM , Wong KHK , Truslow JG , Leung AD , Acharya C , Tien J . Effect of mechanical factors on the function of engineered human blood microvessels in microfluidic collagen gels. Biomaterials. 2010;31:6182‐6189. 20537705 10.1016/j.biomaterials.2010.04.041 PMC2884145

[73] Song JW , Munn LL . Fluid forces control endothelial sprouting. Proc Natl Acad Sci USA. 2011;108:15342‐15347. 21876168 10.1073/pnas.1105316108 PMC3174629

[74] Wong KHK , Truslow JG , Khankhel AH , Chan KLS , Tien J . Artificial lymphatic drainage systems for vascularized microfluidic scaffolds. J Biomed Mater Res A. 2013;101A:2181‐2190. 10.1002/jbm.a.34524 PMC3620968 23281125

[75] DeMaio L , Tarbell JM , Scaduto RC , Gardner TW , Antonetti DA . A transmural pressure gradient induces mechanical and biological adaptive responses in endothelial cells. Am J Physiol Heart Circ Physiol. 2004;286:731‐741. 10.1152/ajpheart.00427.2003 14527936

[76] Galie PA , van Oosten A , Chen CS , Janmey PA . Application of multiple levels of fluid shear stress to endothelial cells plated on polyacrylamide gels. Lab Chip. 2015;15:1205‐1212. 25573790 10.1039/c4lc01236d PMC4500630

[77] Chan KLS , Khankhel AH , Thompson RL , et al. Crosslinking of collagen scaffolds promotes blood and lymphatic vascular stability. J Biomed Mater Res A. 2014;102:3186‐3195. 24151175 10.1002/jbm.a.34990 PMC3995898

[78] Rosenfeld D , Landau S , Shandalov Y , et al. Morphogenesis of 3D vascular networks is regulated by tensile forces. Proc Natl Acad Sci USA. 2016;113:3215‐3220. 26951667 10.1073/pnas.1522273113 PMC4812755

[79] Krishnan L , Underwood CJ , Maas S , et al. Effect of mechanical boundary conditions on orientation of angiogenic microvessels. Cardiovasc Res. 2008;78:324‐332. 18310100 10.1093/cvr/cvn055 PMC2840993

[80] Jiang Y , Torun T , Maffioletti SM , Serio A , Tedesco FS . Bioengineering human skeletal muscle models: recent advances, current challenges and future perspectives. Exp Cell Res. 2022;416:113133. 35427601 10.1016/j.yexcr.2022.113133

[81] Urciuolo A , Serena E , Ghua R , et al. Engineering a 3D in vitro model of human skeletal muscle at the single fiber scale. PLoS One. 2020;15:e0232081. 32374763 10.1371/journal.pone.0232081 PMC7202609

[82] Madden L , Juhas M , Kraus WE , Truskey GA , Bursac N . Bioengineered human myobundles mimic clinical responses of skeletal muscle to drugs. Elife. 2015;4:e04885. 10.7554/eLife.04885 PMC4337710 25575180

[83] Uzel SGM , Platt RJ , Subramanian V , et al. Microfluidic device for the formation of optically excitable, three‐dimensional, compartmentalized motor units. Sci Adv. 2016;2:e1501429. 27493991 10.1126/sciadv.1501429 PMC4972469

[84] Juhas M , Abutaleb N , Wang JT , et al. Incorporation of macrophages into engineered skeletal muscle enables enhanced muscle regeneration. Nat Biomed Eng. 2018;2(12):942‐954. 30581652 10.1038/s41551-018-0290-2 PMC6296488

[85] Takahashi H , Wakayama H , Nagase K , Shimizu T . Engineered human muscle tissue from multilayered aligned myofiber sheets for studies of muscle physiology and predicting drug response. Small Methods. 2023;7:2200849. 10.1002/smtd.202200849 36562139

[86] Zhan RZ , Rao L , Chen Z , Strash N , Bursac N . Loss of sarcomeric proteins via upregulation of JAK/STAT signaling underlies interferon‐γ‐induced contractile deficit in engineered human myocardium. Acta Biomater. 2021;126:144‐153. 33705988 10.1016/j.actbio.2021.03.007 PMC8096718

[87] Nikolić N , Görgens SW , Thoresen GH , Aas V , Eckel J , Eckardt K . Electrical pulse stimulation of cultured skeletal muscle cells as a model for in vitro exercise—possibilities and limitations. Acta Physiol. 2017;220:310‐331. 10.1111/apha.12830 27863008

[88] Rao L , Qian Y , Khodabukus A , Ribar T , Bursac N . Engineering human pluripotent stem cells into a functional skeletal muscle tissue. Nat Commun. 2018;9:126. 29317646 10.1038/s41467-017-02636-4 PMC5760720

[89] Wang J , Broer T , Chavez T , et al. Myoblast deactivation within engineered human skeletal muscle creates a transcriptionally heterogeneous population of quiescent satellite‐like cells. Biomaterials. 2022;284:121508. 35421801 10.1016/j.biomaterials.2022.121508 PMC9289780

[90] Dessauge F , Schleder C , Perruchot MH , Rouger K . 3D in vitro models of skeletal muscle: myopshere, myobundle and bioprinted muscle construct. Vet Res. 2021;52:72. 34011392 10.1186/s13567-021-00942-w PMC8136231

[91] Gillies AR , Chapman MA , Bushong EA , Deerinck TJ , Ellisman MH , Lieber RL . High resolution three‐dimensional reconstruction of fibrotic skeletal muscle extracellular matrix. J Physiol. 2017;595:1159‐1171. 27859324 10.1113/JP273376 PMC5309386

[92] Hinds S , Bian W , Dennis RG , Bursac N . The role of extracellular matrix composition in structure and function of bioengineered skeletal muscle. Biomaterials. 2011;32:3575‐3583. 21324402 10.1016/j.biomaterials.2011.01.062 PMC3057410

[93] Chang ACY , Pardon G , Chang ACH , et al. Increased tissue stiffness triggers contractile dysfunction and telomere shortening in dystrophic cardiomyocytes. Stem Cell Rep. 2021;16:2169‐2181. 10.1016/j.stemcr.2021.04.018 PMC8452491 34019816

[94] Xiang Y , Miller K , Guan J , Kiratitanaporn W , Tang M , Chen S . 3D bioprinting of complex tissues in vitro: state‐of‐the‐art and future perspectives. Arch Toxicol. 2022;96(3):691‐710. 35006284 10.1007/s00204-021-03212-y PMC8850226

[95] Urciuolo A , de Coppi P . Decellularized tissue for muscle regeneration. Int J Mol Sci. 2018;19:2392. 30110909 10.3390/ijms19082392 PMC6121250

[96] Osaki T , Uzel SGM , Kamm RD . Microphysiological 3D model of amyotrophic lateral sclerosis (ALS) from human iPS‐derived muscle cells and optogenetic motor neurons. Sci Adv. 2018;4:eaat5847. 30324134 10.1126/sciadv.aat5847 PMC6179377

[97] Tidball JG , Welc SS . Macrophage‐derived IGF‐1 is a potent coordinator of myogenesis and inflammation in regenerating muscle. Mol Ther. 2015;23:1134‐1135. 26122828 10.1038/mt.2015.97 PMC4817792

[98] Morales MG , Acuña MJ , Cabrera D , Goldschmeding R , Brandan E . The pro‐fibrotic connective tissue growth factor (CTGF/CCN2) correlates with the number of necrotic‐regenerative foci in dystrophic muscle. J Cell Commun Signal. 2018;12:413‐421. 28887614 10.1007/s12079-017-0409-3 PMC5842176

[99] Kim J , Lee J , Kim J , Lee J . Role of transforming growth factor‐β in muscle damage and regeneration: focused on eccentric muscle contraction. J Exerc Rehabil. 2017;13:621‐626. 29326892 10.12965/jer.1735072.536 PMC5747195

[100] Kendall RT , Feghali‐Bostwick CA . Fibroblasts in fibrosis: novel roles and mediators. Front Pharmacol. 2014;5:123. 24904424 10.3389/fphar.2014.00123 PMC4034148

[101] Karvinen H , Pasanen E , Rissanen TT , et al. Long‐term VEGF‐A expression promotes aberrant angiogenesis and fibrosis in skeletal muscle. Gene Therapy. 2011;18(12):1166‐1172. 21562595 10.1038/gt.2011.66

[102] Clere N , Renault S , Corre I . Endothelial‐to‐mesenchymal transition in cancer. Front Cell Dev Biol. 2020;8:569944. 10.3389/fcell.2020.00747 PMC7456955 32923440

[103] Sukriti S , Tauseef M , Yazbeck P , Mehta D . Mechanisms regulating endothelial permeability. Pulm Circ. 2014;4:535‐551. 25610592 10.1086/677356 PMC4278616

[104] Cappellari O , Mantuano P , de Luca A . “The social network” and muscular dystrophies: the lesson learnt about the niche environment as a target for therapeutic strategies. Cells. 2020;9:1659. 32660168 10.3390/cells9071659 PMC7407800

[105] Latroche C , Weiss‐Gayet M , Muller L , et al. Coupling between myogenesis and angiogenesis during skeletal muscle regeneration is stimulated by restorative macrophages. Stem Cell Rep. 2017;9:2018‐2033. 10.1016/j.stemcr.2017.10.027 PMC5785732 29198825

[106] Isobe S , Kataoka M , Endo J , et al. Endothelial‐mesenchymal transition drives expression of CD44 variant and xCT in pulmonary hypertension. Am J Respir Cell Mol Biol. 2019;61:367‐379. 30897333 10.1165/rcmb.2018-0231OC

[107] Gholobova D , Terrie L , Mackova K , et al. Functional evaluation of prevascularization in one‐stage versus two‐stage tissue engineering approach of human bio‐artificial muscle. Biofabrication. 2020;12:035021. 32357347 10.1088/1758-5090/ab8f36

[108] Osaki T , Sivathanu V , Kamm RD . Crosstalk between developing vasculature and optogenetically engineered skeletal muscle improves muscle contraction and angiogenesis. Biomaterials. 2018;156:65‐76. 29190499 10.1016/j.biomaterials.2017.11.041

[109] Bersini S , Gilardi M , Ugolini GS , et al. Engineering an environment for the study of fibrosis: a 3D human muscle model with endothelium specificity and endomysium. Cell Rep. 2018;25:3858‐3868.e4. 30590054 10.1016/j.celrep.2018.11.092

[110] Kizu A , Medici D , Kalluri R . Endothelial‐mesenchymal transition as a novel mechanism for generating myofibroblasts during diabetic nephropathy. Am J Pathol. 2009;175:1371‐1373. 19729485 10.2353/ajpath.2009.090698 PMC2751533

[111] Li J , Qu X , Bertram JF . Endothelial‐myofibroblast transition contributes to the early development of diabetic renal interstitial fibrosis in streptozotocin‐induced diabetic mice. Am J Pathol. 2009;175:1380‐1388. 19729486 10.2353/ajpath.2009.090096 PMC2751535

[112] Hughes DP , Marron MB , Brindle NPJ . The antiinflammatory endothelial tyrosine kinase Tie2 interacts with a novel nuclear factor‐κB inhibitor ABIN‐2. Circ Res. 2003;92:630‐636. 12609966 10.1161/01.RES.0000063422.38690.DC

[113] Kim H , Osaki T , Kamm RD , Asada HH . Tri‐culture of spatially organizing human skeletal muscle cells, endothelial cells, and fibroblasts enhances contractile force and vascular perfusion of skeletal muscle tissues. FASEB J. 2022;36:e22453. 35838893 10.1096/fj.202200500R PMC12166285

[114] Kim H , Osaki T , Kamm RD , Asada HH . Multiscale engineered human skeletal muscles with perfusable vasculature and microvascular network recapitulating the fluid compartments. Biofabrication. 2022;15:015005. 10.1088/1758-5090/ac933d 36126639

[115] Paik DT , Tian L , Williams IM , et al. Single‐cell RNA sequencing unveils unique transcriptomic signatures of organ‐specific endothelial cells. Circulation. 2020;142:1848‐1862. 32929989 10.1161/CIRCULATIONAHA.119.041433 PMC7658053

[116] Arrigoni C , Ostano P , Bersini S , et al. Differential angiogenesis of bone and muscle endothelium in aging and inflammatory processes. Commun Biol. 2023;6:126. 36721025 10.1038/s42003-023-04515-9 PMC9889796

[117] Bersini S , Francescato R , Moretti M . Biofabrication of 3D human muscle model with vascularization and endomysium. Methods Mol Biol. 2022;2373:213‐230. 34520015 10.1007/978-1-0716-1693-2_13

[118] Kahaleh B . Vascular disease in scleroderma: mechanisms of vascular injury. Rheum Dis Clin North Am. 2008;34:57‐71. 18329532 10.1016/j.rdc.2007.12.004

[119] Kramer B , Corallo C , van den Heuvel A , et al. High‐throughput 3D microvessel‐on‐a‐chip model to study defective angiogenesis in systemic sclerosis. Sci Rep. 2022;12(1):16930. 36209279 10.1038/s41598-022-21468-x PMC9547891

[120] Whiteford J , Arokiasamy S , Thompson CL , Dufton NP . Novel application of live imaging to determine the functional cell biology of endothelial‐to‐mesenchymal transition (EndMT) within a liver‐on‐a‐chip platform. In Vitro Models. 2022;1(6):413‐421. 36570669 10.1007/s44164-022-00034-9 PMC9767233

[121] Bramsen JA , Alber B , Murray B , Chen MH , Huang P , Mahler G . Endothelial to mesenchymal transformation‐derived activated fibroblast behavior in a 3D culture environment. Struct Heart. 2021;5:21.

[122] Abdalkader R , Konishi S , Fujita T . The development of biomimetic aligned skeletal muscles in a fully 3d printed microfluidic device. Biomimetics. 2022;7:2. 10.3390/biomimetics7010002 PMC8788470 35076457

[123] Wang J , Zhou CJ , Khodabukus A , et al. Three‐dimensional tissue‐engineered human skeletal muscle model of Pompe disease. Commun Biol. 2021;4:524. 33953320 10.1038/s42003-021-02059-4 PMC8100136

[124] Vila OF , Chavez M , Ma SP , et al. Bioengineered optogenetic model of human neuromuscular junction. Biomaterials. 2021;276:121033. 34403849 10.1016/j.biomaterials.2021.121033 PMC8439334

[125] Widyantoro B , Emoto N , Nakayama K , et al. Endothelial cell–derived endothelin‐1 promotes cardiac fibrosis in diabetic hearts through stimulation of endothelial‐to‐mesenchymal transition. Circulation. 2010;121:2407‐2418. 20497976 10.1161/CIRCULATIONAHA.110.938217

[126] Hashimoto N , Phan SH , Imaizumi K , et al. Endothelial–mesenchymal transition in bleomycin‐induced pulmonary fibrosis. Am J Respir Cell Mol Biol. 2010;43:161‐172. doi:10.1165/rcmb.2009-0031OC 19767450 PMC2937229

[127] Maddaluno L , Rudini N , Cuttano R , et al. EndMT contributes to the onset and progression of cerebral cavernous malformations. Nature. 2013;498(7455):492‐496. 23748444 10.1038/nature12207

[128] Zordan P , Rigamonti E , Freudenberg K , et al. Macrophages commit postnatal endothelium‐derived progenitors to angiogenesis and restrict endothelial to mesenchymal transition during muscle regeneration. Cell Death Dis. 2014;5(1):e1031. 24481445 10.1038/cddis.2013.558 PMC4040684

[129] Tirone M , Giovenzana A , Vallone A , et al. Severe heterotopic ossification in the skeletal muscle and endothelial cells recruitment to chondrogenesis are enhanced by monocyte/macrophage depletion. Front Immunol. 2019;10:1640. 31396210 10.3389/fimmu.2019.01640 PMC6662553

[130] Grenier G , Scimè A , le Grand F , et al. Resident endothelial precursors in muscle, adipose, and dermis contribute to postnatal Vasculogenesis. Stem Cells. 2007;25:3101‐3110. 17823241 10.1634/stemcells.2006-0795

[131] Alonso‐Herranz L , Sahún‐Español Á , Paredes A , et al. Macrophages promote endothelial‐to‐mesenchymal transition via MT1‐MMP/ TGFβ1 after myocardial infarction. Elife. 2020;9:1‐30. 10.7554/eLife.57920 PMC7609061 33063665

[132] Lamb YN . Nintedanib: a review in fibrotic interstitial lung diseases. Drugs. 2021;81:575‐586. 33765296 10.1007/s40265-021-01487-0 PMC8163683

[133] Sathiyamoorthy G , Sehgal S , Ashton RW . Pirfenidone and Nintedanib for treatment of idiopathic pulmonary fibrosis. South Med J. 2017;110:393‐398. 28575896 10.14423/SMJ.0000000000000655

